# The New York Institute of Stomatology

**Published:** 1898-02

**Authors:** 

**Affiliations:** The New York Institute of Stomatology


### THE NEW YORK INSTITUTE OF STOMATOLOGY.

A regular meeting of the Institute was held Friday evening, November 5, 1897, at the office of Dr. George S. Allan, No. 51 West Thirty-seventh Street, the President, Dr. George S. Allan, in the chair.

The minutes of the previous meeting were read and approved.

COMMUNICATIONS ON THEORY AND PRACTICE.

*Dr. S. F. Howland.*—I would like to know what has been the experience of those present as to the length of time a retaining appliance should be worn to keep teeth in proper position after regulation. In the case I have in mind, the superior incisors were moved backward, the centrals being moved nearly a half-inch. I once had a similar case where the retaining plate was worn a year, and proved insufficient, as the teeth moved slightly after its removal. In the case I now mention the teeth have been kept in place one year, and I propose keeping the retaining plate in at least six months longer.

*Dr. J. F. P. Hodson.*—I do not think there is anything in the way of regulating teeth that is so unsatisfactory as moving back upper central incisors, or, rather, keeping them in position thereafter by any ordinary method of limited rotation. I have, therefore, invariably made cavities in the posterior portions of the teeth, and put a little band of gold inside the front teeth, attached to tiny studs set into the cavities, to stay there for an almost indefinite period. I do not think that the tendency of the upper front teeth to move forward again can be overcome in any single year or two. I keep them back for a series of years with a positive appliance.

*The President.*—May I ask Dr. Hodson to explain more fully his method.

*Dr. Hodson.*—I would deliberately make cavities in the posterior surface—there are natural depressions there already—for the purpose of forming sufficient holding points for the tiny studs attached to the half-round wire. This wire would extend along the gum inside and be attached to some tooth on each side of the mouth back of the teeth to be retained. The little studs, being cemented into the cavities, would hold the teeth positively, and the gold, not showing in front, would be worn without protest for a long enough period to make me sure that the teeth would not move from their new positions.

*Dr. C. A. Woodward.*—I have heard reports of disagreeable results in taking X-ray photographs, one of which may be of interest. A lady came from the West to have me diagnose her case, and if possible relieve her. She had had trouble in the region of the antrum for nearly fifteen years. On her arrival in the city, finding that I was out of town, she went to a surgeon for advice. He examined her mouth, and found that all of the molars and bicuspids had been removed on that side from time to time, as it was thought that they might have been the cause of the trouble. He could see nothing that was abnormal, but, thinking it possible that there might be an undeveloped tooth, advised her to have an X-ray photograph taken. He sent her to one of the most skilful men in the city, and while having the photograph taken she experienced no particular discomfort or pain. The time consumed in taking the photograph was about ten minutes.

On the morning of the second or third day after the photograph had been taken, while she was arranging her hair, all the hair on that side of her head came out. The next morning she found her face was deeply wrinkled, the flesh drawn and very red. She became alarmed, and consulted one of the best-known physicians in town, telling him what had been done. He advised her not to leave town at once, as she desired to do, but said that it was absolutely necessary that she should return to her hotel and shut herself up in her room for at least twelve days for if she did not he would not be responsible for the result. I will say that he feared that the inflammation might go to the ear, and also to her eye. At the end of twelve or fifteen days she was obliged to leave the city. Before going, she thought she would call upon the gentleman who took the photograph, to see what explanation he could make. He said he was very sorry, and presumed that he had held the lamp too close to her face.

*Dr. S. F. Wilcox.*—I happen to know something of the bad results of the X-rays, because I have seen such results in two of my cases. When the X-ray was first discovered, it was supposed to be entirely harmless, and so it was used considerably, especially for finding bullets, and at the Flower Hospital there were some severe burns resulting from its use. I happened to hear of a very persistent case of ostitis, which had been under the care of the best surgeons in Philadelphia. The man had some trouble with his thumb, and, in order to determine what it was, one of the surgeons asked him to go to the laboratory and have it photographed. They could find nothing which showed particularly, but the result of the application of the rays to the bone was that it immediately got well. So, having two cases of ostitis of the tibia, I thought I would try the X-rays on them The rays were applied by a skilful electrician, and the relief from pain in the bone was something remarkable. In one case the patient had complete relief for quite a long time, but the pain afterwards returned. In the other case the relief was not quite so marked. In the first case the patient had an attack of paralysis on one side, and was confined to his bed, and while in bed the pain returned and was exceedingly severe, so it was thought best to try the X-rays again. The doctor had a static battery set up in the patient's room, and the lamp was not placed directly over the leg, but a short distance from it; there was not, however, so much relief this time as on the former occasion. The pain and tension became so great that I cut down through the tibia into the medullary canal, and this relieved the bone pain at once. At the time of the operation there was only a dark discoloration of the skin. Shortly afterwards, while the healing process was going on in the bone, the skin began to come off at the dark spot, which was about as large as my hand, and the part was entirely denuded of skin, leaving an irritable, indolent ulcer, which up to this time has utterly refused to heal.

The X-rays not only destroy the skin, but seem to prevent all power of repair. They also seem to destroy all vitality in the subcutaneous tissue,—not exactly kill it, but destroy all power of reproduction of new cells. In this case the patient's general condition was low, but still, even with improved health, there is no improvement in the local condition. It is claimed that this is due to the fact that ozone is generated and has an irritating effect on the tissues when the lamp is held close.

Somebody, a short time ago, said that he had invented some sort of an arrangement which would prevent the destructive action of the X-rays, but it is impossible to get it from any one but himself, and, as his price is so high, it is hardly known whether it is a "fake" or not.

*The President.*—Do X-rays produce local paralysis?

*Dr. Wilcox.*—No, they do not produce paralysis, but a destruction of tissue and an apparent inhibition of all proliferative power in the tissues.

*The President.*—Does it affect the nerves?

*Dr. Wilcox.*—No, I do not think so. It is claimed by some electricians that the trouble is not produced by the use of the static machine; but that is a mistake, because in the cases I spoke of the trouble was all produced by that.

The paper of the evening, entitled "The Philosophy of Exudative Inflammation," was then read by its author, John Rogers, M.D., who also showed charts prepared by Dr. Ewing, illustrating the paper.

(For Dr. Rogers's paper, see page 65.)

DISCUSSION.

*The President.*—Desiring to bring this subject before the society in all its latest developments, I asked Dr. Rogers if he would prepare this paper, so that we could have the story of inflammation, and the processes attending it, fairly before us. When I first spoke to Dr. Rogers, I hoped to have the paper divided into two portions, —the first being the theory of inflammation, and the second, the clinical aspects of it. But I found that the subject was by far too large to be condensed into the time that could be devoted to it.

As to the thoroughness with which Dr. Rogers has completed his task, I think you are all ready to say that he has covered the ground, and I now take pleasure in asking you to discuss the matter fully.

*Dr. Wilcox.*—From the beginning to the end of the excellent and comprehensive way in which Dr. Rogers has treated the subject, there is nothing left to discuss. The whole ground has been covered. I cannot see a single point to bring up any further than the doctor has done.

Most of our theory of inflammation has been based upon the discoveries of Cohnheim. He, of course, made his experiments and published them before the present methods of aseptic surgery were known. It has always seemed to me that the way in which he made his experiments must have been accompanied by more or less infection. I would like to ask Dr. Rogers the method in which the aseptic reparative process is now demonstrated. I mean, can there be any possibility of infection in any way, by the atmosphere or anything else?

*Dr. John Rogers.*—The aseptic repair, I believe, is demonstrated by making several wounds in the same rabbit, or several rabbits, and keeping them carefully clean and aseptic, then killing the rabbits at different stages, and making sections of the results. There is always possibility of infection of the wound, but a wound can be made aseptically and kept aseptic.

*Dr. James Ewing.*—I do not know that I can add very much to Dr. Rogers's explanation of the charts. I may say that one is intended to illustrate what was originally known as the "Phenomenon of Pfeiffer,"—that when certain bacteria are injected into the peritoneal cavity of immune animals, the bacteria are destroyed with most remarkable rapidity.

In examining the phagocytic cells in this experiment, at early periods after the injection into the immune animal, the bacteria were first found ingested by the different forms of leucocytes, and in examining these leucocytes very shortly after the disappearance of the bacteria from the peritoneal cavity, Pfeiffer found them in all stages of granular disintegration. He followed this degeneration still further, and found that the ordinary staining reaction was lost very shortly after the bacteria were absorbed into the leucocytes, that they stained with red acid dyes instead of the blue basic dyes, and finally began to lose staining capacity entirely.

You are all familiar with the recent application of the serum of immunized animals in cases of diphtheria. It is the first practical application of the bactericidal properties of body fluids observed in the "Phenomenon of Pfeiffer," in general medicine. In Widal's test in typhoid fever, also, we see the action of a serum of an immune patient upon the actively motile bacilli, in collecting them first, causing loss of motility, and finally breaking them up into small granules.

As to the other charts, I have nothing to say. They have been fully elucidated.

The views which are given by Dr. Rogers are those generally accepted, though not entirely so, by the English school, but not by the German.

The classification of leucocytes, also, has not been thoroughly worked out, nor can it be said that all the functions of the different leucocytes are so closely identified with the different parts of the process of immunity as some Englishmen would have us believe. The simple observation that soon after the injection the bacteria are found within the leucocytes would not be accepted by the average pathologist as positive proof that this process results entirely from the action of the leucocytes.

I have been struck by the improvement and the advance made in the study of inflammation in the last ten years. Fifteen years ago we would have had to be content almost entirely with the mechanical side of the question. We could show how blood-vessels were injured and serum was exuded, and could follow the fate of these exuded products into the repair or necrosis of the tissues, and there we would have to stop. But thanks to the study of many eminent pathologists, we have an entirely different side of the subject as shown in Dr. Rogers's paper, and there is no question but that that is the essential part of the process. In these charts I have endeavored to show some of the evidences that the chemical process is at the base of most previously considered mechanical processes of inflammation.

*Dr. J. Morgan Howe.*—If I understood correctly, Dr. Rogers said, in his paper, that phagocytosis is rather a secondary germicidal property, and that the germicidal properties of the blood-serum are rather more important.

*Dr. Rogers.*—I think that is the general consensus of opinion of most pathologists, that phagocytosis is secondary.

Metschnikoff, who was practically the originator of the theory of phagocytosis, believed that everything was dependent upon it; but there are too many experiments to show that the bacteria are dead before phagocytosis has occurred, to make that entirely tenable. Nevertheless, it is probable that the bactericidal substances are ferments, and as such are derived from the cells. The one process can hardly be said to be more important than the other, inasmuch as the extra- and intracellular micro-organisms are affected by the same substances. Those outside the cells are injured by the secretion, so to speak, which digests and so destroys those which have been devoured.

*Dr. Ewing.*—I would like to say that, although every one is aware that phagocytosis is a secondary process in inflammation, the services of Metschnikoff were not so much in calling attention to the process of phagocytosis, as in demonstrating the importance of the leucocytes in inflammation. The fact that the ingestion of the bacteria may be preceded by other charges does not detract from the value of his observations. He has shown that the leucocytes are essential in the process of immunity, and the only addition to his work is that the immunizing substances are produced by the leucocytes, and in some instances it is necessary for them to make use of these immunizing substances, which destroy the virulence of bacteria before phagocytosis occurs, the leucocytes, nevertheless, being the chief agent in the process.

Adjourned.

S. E. DAVENPORT, D.D.S., M.D.S.,
*Editor The New York Institute of Stomatology.*

